# STAT3 governs hyporesponsiveness and granzyme B-dependent suppressive capacity in human CD4^+^ T cells

**DOI:** 10.1096/fj.14-257584

**Published:** 2014-11-14

**Authors:** Klaus G. Schmetterer, Alina Neunkirchner, Daniela Wojta-Stremayr, Judith Leitner, Peter Steinberger, Winfried F. Pickl

**Affiliations:** *Institute of Immunology, Center for Pathophysiology, Infectiology and Immunology, Medical University of Vienna, Vienna, Austria; ^†^Christian Doppler Laboratory for Immunomodulation, Vienna, Austria; and ^‡^Department of Laboratory Medicine, Medical University of Vienna, Vienna, Austria

**Keywords:** regulatory T cells, Tr1 cells

## Abstract

Signal transducer and activator of transcription 3 (STAT3) integrates key signals of cell surface immune receptors, yet its precise role in cluster of differentiation (CD)4^+^ T cells is not well-established. Current research has indicated T-helper cell 17–inducing roles but also tolerogenic roles. To address this issue, human T cells were transduced with the constitutively active STAT3 mutant *STAT3C*. Following stimulation, STAT3C^+^ T cells up-regulated IL-10 (4.1 ± 0.5-fold; *P* < 0.001) and granzyme B (2.5 ± 1.2, *P* < 0.05) secretion, combined with significantly reduced IFN-*γ* (35 ± 5%), IL-2 (57 ± 4%), TNF-*α* (64 ± 8%), and IL-13 (89 ± 3%) secretion (*P* < 0.001). CD3/CD2- or CD3/CD28-activated STAT3C^+^ T cells revealed reduced proliferation (53.4 ± 23.5% and 70.5 ± 10.4%, respectively), which was independent of IL-10 production and significantly suppressed effector T cell proliferation by 68.7 ± 10.6% and 65.9 ± 2.6%, respectively (*P* < 0.001). Phenotypically, *STAT3C*-transgenic CD4^+^ T cells resembled effector T cells regarding expression of T regulatory cell markers, but up-regulated granzyme B expression levels by 2.4-fold (*P* < 0.05). Suppression was cell contact dependent and mediated by granzyme B-induced cell death, but was independent of IL-10 and TGF-*β*. Notably, peripheral blood CD4^+^CD45RA^−^lymphocyte activation gene-3^+^CD49^+^ type 1 regulatory T cells revealed activation-induced hyperphosphorylation of STAT3. In agreement, pharmacological inhibition of STAT3 activation partially reverted hyporesponsiveness of peripheral type 1 regulatory T cells (increasing their division index from 0.46 ± 0.11 to 0.89 ± 0.04; *P* < 0.01). These observations indicate a clear-cut relation between activation of STAT3 and the acquisition of a tolerogenic program, which is also used by peripheral blood type 1 regulatory T cells.—Schmetterer, K. G., Neunkirchner, A., Wojta-Stremayr, D., Leitner, J., Steinberger, P., Pickl, W. F. STAT3 governs hyporesponsiveness and granzyme B-dependent suppressive capacity in human CD4^+^ T cells.

The signal transducer and activator of transcription 3 (STAT3) is a key molecule in the integration of signals derived from receptor tyrosine kinases and nonreceptor tyrosine kinases. Similar to other STAT proteins, phosphorylation of STAT3 leads to dimerization of the cytosolic protein followed by translocation to the nucleus, where STAT3 dimers act as transcription factors. Because of its highly pleiotropic role in different tissues, STAT3 is involved in a multitude of physiologic and pathophysiologic processes ranging from embryogenesis and immunoregulation to carcinogenesis.

In myeloid immune cells, both loss-of-function (1–4) and overexpression studies (3, 5) and studies in human dendritic cells (6) have confirmed that activation of STAT3 mainly governs anti-inflammatory and tolerogenic processes. In contrast, the role of STAT3 in lymphocytes appears to be more ambiguous. Conditional knockout of *Stat3* in cluster of differentiation (CD)4^+^ T cells completely abrogates their ability to differentiate into T-helper (Th)17 cells. Reversely, overexpression of a constitutively active form of STAT3, termed STAT3C, was shown to strongly induce Th17 polarization in murine T cells (7–9), which is governed by the upstream activity of PKC-*θ* (10). Interestingly, the Th17-inducing capacity of STAT3C was not consistently found but only observed in the absence of IFN-*γ*, identifying IFN-*γ* as a potential antagonist of Th17 polarization (8).

Conversely, potential tolerogenic aspects in CD4^+^ T cells have been highlighted as the second major function of STAT3 signaling in recent reports (11–13). Remarkably, deletion of *Stat3* in CD4^+^CD25^+^ naturally occurring T regulatory cells (nTreg) impaired their ability to suppress Th17 responses (11), which could subsequently be defined as an IL-10-dependent process (12). Similarly, pharmacological or siRNA-mediated inhibition of STAT3 decreased conversion of CD4^+^CD25^−^ T cells into regulatory T cells (13). Previous reports also suggested that tolerogenic aspects of STAT3 might play an important role in the induction and function of IL-10-secreting type 1 regulatory T cells (Tr1). These cells are marked by a typical cytokine secretion profile including high levels of IL-10, intermediate levels of IFN-*γ*, and low levels of IL-2 and IL-4 (14). Tr1 cells can be induced *in vitro* by different protocols including stimulation with immature dendritic cells (15), IL-10 and/or IFN-*α* (16), and IL-27 (17–20), all inducing STAT3 signaling in target T cells [reviewed by Gregori *et al.* (21)]. The recent identification of CD4^+^CD45RA^−^lymphocyte activation gene-3 (LAG3)^+^CD49b^+^ phenotype as a specific cell surface marker combination for human peripheral blood (PB) Tr1 cells (22) offers the possibility to separate these cells from PB and to study their biology in a more unbiased way without the need for prior induction from nonregulatory T cells. However, to the best of our knowledge, the activation status of STAT3 in these cells has thus far not been examined.

The 2 roles of STAT3 are probably best reflected by the pathophysiology caused by autosomal-dominant STAT3 mutations in hyper IgE syndrome. In this disease, patients are deficient for Th17 cells but also display typical signs and symptoms of immune dysregulation, such as IgE hyperproduction and eczema, both of which are typically associated with other well-described loss-of-tolerance diseases such as immunodeficiency, polyendocrinopathy, enteropathy, X-linked syndrome [forkhead box protein 3 (FOXP3) mutations], autoimmune, polyendocrinopathy, candidiasis, ectodermal dystrophy syndrome (autoimmune regulator mutations), and Omenn’s syndrome (recombination-activating gene mutations) (23).

To elucidate the functional role of STAT3 in human CD4^+^ T cells, we ectopically expressed a constitutively active form of STAT3, designated STAT3C (24), in PB CD4^+^ T cells of healthy human individuals. *STAT3C*-transduced T cells were analyzed for their phenotype in regard to nTreg and Tr1 markers. We also assessed their proliferation and cytokine production potential following T cell receptor–mediated activation in association with different costimulatory signals. Furthermore, their suppressive capacity was investigated in cocultures with effector T cells in response to various forms of antigen-presenting cells (APCs) and compared with *FOXP3*-transduced T cells, which represent in functional terms *bona fide* nTreg cells (25–27). Finally, we correlated the results obtained in overexpression studies with the activation status of STAT3 in resting and activated human PB Tr1 cells in comparison with effector PB T cells and assessed the influence of STAT3 activation on the proliferative capacity of Tr1 cells.

## MATERIALS AND METHODS

### Molecular cloning and generation of multicistronic vectors

The *STAT3* cDNA was amplified from a human T cell cDNA library (28) with the following primers: STAT3 forward, 5′-CCCGCG**AAGCTT**GCCACCATGGCCCAATGGAATCAGCTACAGC-3′; STAT3 reverse, 5′-CCCGCG**GCGGCCGC**TTTACATGGGGGAGGTAGCGCACTC-3′; STAT3Cint forward, 5′-ATGGGCTATAAGATCATGGATTGCACCTGCATCCTGGTGTCTCCACTG-3′; STAT3Cint reverse, 5′-CAGTGGAGACACCAGGATGCAGGTGCAATCCATGATCTTATAGCCCAT-3′ (bold sequences mark restriction enzyme sites). The *STAT3(p.A661C, p.N663C)* (in the following referred to as *STAT3C*) cDNA was generated from the wild-type *STAT3* construct *via* PCR mutagenesis. Both constructs were digested with *Hind*III and *Not*I and ligated into the retroviral pMMP-internal ribosomal entry site (IRES)-green fluorescent protein (GFP) vector. The pMMP-FOXP3-IRES-GFP and empty control pMMP-IRES-GFP vector were described elsewhere (27).

### Cell lines and primary cells

The HEK-293 cell line (human embryonic kidney cells) was cultured as described previously (29). Peripheral blood mononuclear cells were isolated from healthy volunteers in compliance with the ethics committee of the Medical University of Vienna. CD4^+^ T cells were isolated using the Human CD4^+^ T cell Isolation Kit II (Miltenyi Biotech, Bergisch Gladbach, Germany). Human CD14^+^ monocytes were isolated using CD14 MicroBeads (Miltenyi Biotech). For generation of monocyte-derived dendritic cells (mdDCs), CD14^+^ monocytes were cultured for 7 d with GM-CSF and IL-4. Some cultures were additionally supplemented with 100 ng/ml LPS in the final 48 h of culture to generate mature mdDCs. The mouse thymoma cell line BW5417 expressing a membrane-bound OKT3 single-chain variable fragment (scFv) (BW 3/2) in concert with the costimulatory molecule CD58 was cultured and maintained as described previously (30).

### Transfection of HEK-293 cells

HEK-293 cells were transfected using Ca_2_PO_4_ precipitation method as described previously (29). For the production of amphotropic T cell–transducing retrovirus supernatants, MoMLV *gag-pol*, the envelope protein GalV and transgene DNA were transfected in a 2:1:3 ratio.

### Retroviral transduction of peripheral blood T cells

CD4^+^ PB T cells (5 × 10^6^/well) were stimulated in 6-well flat bottom plates with 5 × 10^6^ anti-CD3/CD28 coated microbeads (Dynabeads, Invitrogen, Carlsbad, CA, USA) and 300 U/ml IL-2 (Peprotech, London, United Kingdom) for 48 h. Retroviral transduction was performed by addition of cell-free retroviral supernatant in the presence of 8 *µ*g/ml polyprene (Sigma-Aldrich, St. Louis, MO, USA) followed by centrifugation at 600 *g* for 2 h. Twenty-four hours after transduction, cells were transferred to fresh medium containing 100 U/ml IL-2 and cultured for another 6–7 d (31).

### Flow cytometric analyses

Cells were stained as described previously (32) using the mAbs indicated in Supplemental Table 1 (20 *µ*g/ml). For intracellular staining of the transcription factors FOXP3 and STAT3, the FOXP3 Fix/Perm Buffer Set (BioLegend, San Diego, CA, USA) was used according to the manufacturers’ recommendations. For intracellular staining of granzyme A, granzyme B, and cytotoxic T-lymphocyte antigen (CTLA)-4, the Fix and Perm Kit from ADG (An der Grub, Kaumberg, Austria) was used. For intracellular staining of phosphorylated STAT3, the BD Phosflow Fixation Buffer I, Permeabilization Buffer III system (BD Biosciences, Franklin Lakes, NJ, USA) was used according to the manufacturer’s instructions. Analyses were performed on a BD LSRII Fortessa flow cytometer using the FlowJo software (BD Biosciences).

### Determination of cytokine secretion

Transduced T cells (5 × 10^4^) were stimulated in 96-well plates with either the BW 3/2 CD58 cell line serving as T cell stimulator cells (2 × 10^4^ irradiated with 60 Gy) or anti-CD3/CD28 coated microbeads (5 × 10^4^). After 24, 48, 72, or 96 h, supernatants were harvested, and cytokine concentrations were determined by multiplex analysis (Luminex 100IS; Biomedica, Vienna, Austria). For cytokine analyses of mixed lymphocyte reaction coculture experiments, cytokines were harvested after 120 h and processed as above.

### Determination of granzyme B secretion

Transduced T cells (1 × 10^5^) were stimulated in 96-well plates with either with BW 3/2 CD58 cells (4 × 10^4^ irradiated with 60 Gy) or anti-CD3/CD28 coated microbeads (1 × 10^5^). After 6 h, supernatants were harvested, and granzyme B concentrations were determined using the granzyme B Platinum ELISA (eBioscience, San Diego, CA, USA) according to the manufacturer's instructions.

### Proliferation and suppression assays

Transduced T cells (5 × 10^4^) were stimulated in 96-well plates with BW 3/2 CD58 artificial APCs (aAPCs; 2 × 10^4^ irradiated with 60 Gy) or anti-CD3/anti-CD28 coated microbeads at the indicated cell to bead ratios for 72 h, pulsed with [methyl-^3^H] thymidine (1 *µ*Ci per well; Perkin Elmer, Boston, MA, USA) for an additional 18 h, and processed as described previously (29). To test the suppressive capability of transgenic T cells, 5 × 10^4^ effector T cells were cocultured with flow cytometrically sorted STAT3C^+^, FOXP3^+^, or control-transduced T cells and stimulated with aAPCs or anti-CD3/anti-CD28 coated microbeads and processed as above. Similarly, CD4^+^ effector T cells were labeled with the far-red cell proliferation dye eFluor 670 (eBioscience; 1.5 *µ*M), cocultured with transduced T cells, and analyzed by flow cytometry after 96 h. In some experiments, blocking antibodies to IL-10 (3C12C12; Santa Cruz Biotechnology, Heidelberg, Germany), TGF-*β*1,2,3 (1D11; R&D Systems, Minneapolis, MN, USA), or control mouse IgG_1_ (11711; R&D Systems) were added at a final concentration of 20 *µ*g/ml in accordance with established methods (33–35). Similarly, in some experiments, the granzyme B-specific inhibitor benzyloxycarbonyl-ala-ala-asp(OMe) chloromethyl ketone (Z-AAD-CMK; granzyme B inhibitor I; Merck Millipore, Darmstadt, Germany) was added to the cocultures at a final concentration of 30 *µ*M (36).

For allogeneic mixed lymphocyte reactions, 5 × 10^4^ CD4^+^ T cells were cocultured with fluorescence-activated cell sorted (FACS) *STAT3C*-, *FOXP3*-, or control-transduced T cells and stimulated with allogeneic immature and LPS-matured mdDCs (5 × 10^3^) in 96-well round-bottom plates for 120 h. Cultures were pulsed with [methyl-^3^H] thymidine for an additional 18 h and processed as above.

For transwell analyses, 2 × 10^5^ control-transduced or STAT3C^+^ T cells were seeded into the lower bucket of a transwell system (pore size, 3 *µ*m; Corning Costar, Corning, NY, USA) and stimulated with 1 × 10^5^ BW 3/2 CD58 aAPCs or 1 × 10^5^ anti-CD3/anti-CD28 coated microbeads; 5 × 10^4^ eFluor670 CPD-labeled CD4^+^ T cells were seeded into the upper well and stimulated with 2 × 10^4^ BW 3/2 CD58 aAPCs. After 96 h, proliferation of the CD4^+^ responder T cells from the upper well was assessed by flow cytometry as above.

For assessment of the influence of STAT3 activation on Tr1 cell proliferation, magnetic cell isolation and cell separation CD4^+^ T cells were labeled with the eFluor450 cell proliferation dye (eBioscience; 1 *µ*M), rested overnight in medium before FACS sorting for CD4^+^CD45RA^−^CD49b^+^LAG-3^+^ Tr1 and CD4^+^CD45RA^−^CD49b^−^LAG-3^−^ effector T cell (T_EFF_) cells. T cell populations (5 × 10^4^) were activated in a 96-well flat-bottom plates using 5 × 10^4^ anti-CD3/anti-CD28 coated microbeads in the presence or absence of 100 *µ*M STAT3 inhibitor V (STAT Three Inhibitory Compound; Merck, Whitehouse Station, NJ, USA) (37). After a 96 h dilution of eFluor450, Cell Proliferation Dye was determined as a measure for cellular proliferation by flow cytometry.

### Determination of cell death

CD4^+^ T cells (5 × 10^4^) were cocultured in the presence or absence of 5 × 10^4^ control vector- or STAT3C-transduced T cells in 96-well flat-bottom plates and activated using 2 × 10^4^ BW 3/2 CD58 aAPCs. After 72 h, cells were harvested and washed in annexin staining buffer (1 mM [4-(2-hydroxyethyl)-1-piperazineethanesulfonic acid], 140 mM NaCl, and 25 *µ*M CaCl_2_; all Sigma-Aldrich). After addition of 5 *µ*l annexin V APC (eBioscience), cells were incubated at room temperature for 10 min. Immediately prior to analysis, propidium iodide (50 ng/ml; Sigma-Aldrich) was added. Cell death of GFP-negative T_EFF_ was determined by flow cytometry by quantification of the cumulative percentage of cells positive for annexin V and/or propidium iodide. Numbers were then normalized to the degree of cell death observed in T_EFF_ activated in plain medium, which amounted to 45.1 ± 1.5%.

### Statistical analyses

For comparison of proliferation data from CPD-labeled effector T cells, a division index was calculated according to the formula division index = [log(mean fluorescence nonstimulated cells/mean fluorescence responder cells)/log^2^] (38). For multiple group comparisons, 1-way ANOVA followed by Bonferroni correction was performed using GraphPad Prism version 5.0 for Windows (GraphPad Software, San Diego CA, USA). Two-group comparisons were performed using the Student’s *t* test. Data represent mean values ± sd. Statistically significant values are denoted as follows: *P < 0.05; **P < 0.01; and ***P < 0.001.

## RESULTS

### Generation and phenotyping of human STAT3^+^ and STAT3C^+^ CD4^+^ T cells

**Figure 1.**
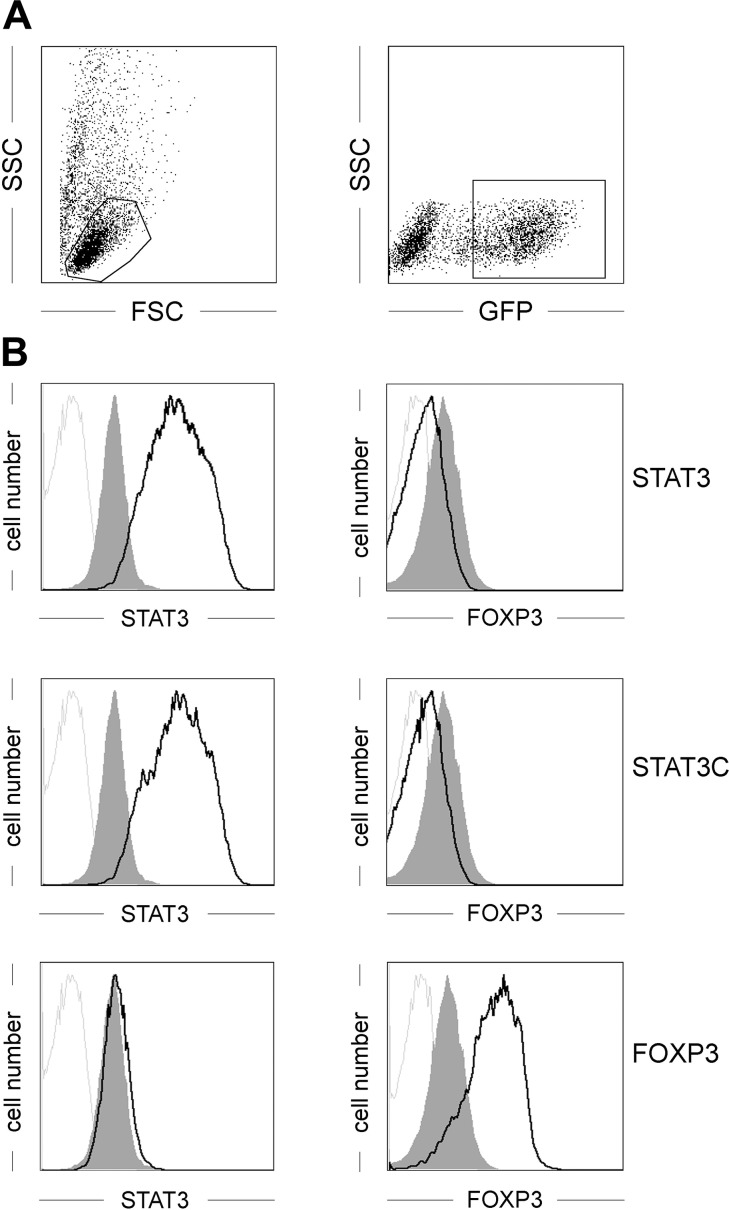
Intracellular expression pattern in CD4^+^ T cells of transcription factors. Primary human CD4^+^ T cells were retrovirally transduced either with human *STAT3, STAT3C, FOXP3,* or an empty control vector and analyzed for expression of the indicated transcription factors. *A*) Gating strategy for positively transduced T cells. *B*) Intracellular expression levels of the indicated transcription factors in control vector-transduced cells (gray histograms) or transcription factor-transduced cells (black lines) using directly labeled mAbs. Gray lines depict staining with isotype-matched control mAb. One representative experiment of 8 is depicted.

Human *STAT3* was cloned from a human dendritic cell cDNA library (39) and modified by PCR mutagenesis to generate the *STAT3 (p.A661C, p.N663C)* mutant (termed STAT3C), which has been described to act as a constitutively active form of STAT3 (40). Both constructs were cloned into the retroviral pMMP-IRES-GFP vector (27), which enables efficient transduction and monitoring of transgene expression (**Fig. 1*A***). Following retroviral transduction, *STAT3*-transduced and *STAT3C*-transduced, but not *FOXP3*-transduced, CD4^+^ T cells showed a clear-cut overexpression of STAT3 compared with endogenous expression levels (Fig. 1*B*). Concomitant analysis for intracellular expression of the transcription factor FOXP3, which is the key mediator of nTreg development and tolerogenic function (41, 42), showed no up-regulation in *STAT3*-transduced or *STAT3C*-transduced T cells (Fig. 1*B*), but was found clearly expressed in *FOXP3*-transduced T cells.

### STAT3C^+^ T cells are hyporesponsive to TCR-mediated stimulation

**Figure 2.**
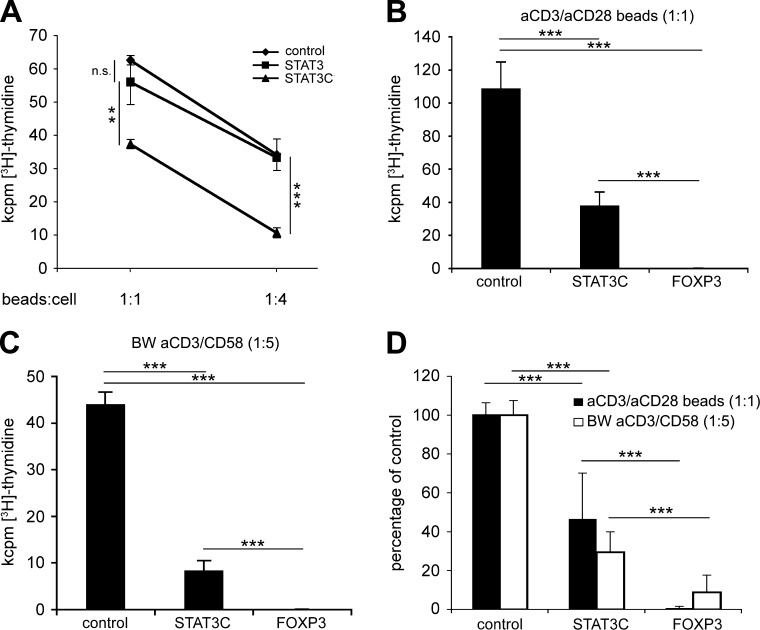
*STAT3C*-transduced CD4^+^ T cells are hyporesponsive. *A*) Proliferation of STAT3^+^, STAT3C^+^, or control-transduced T cells in response to anti-CD3/anti-CD28 coated microbeads at the indicated bead to cell ratio. *B*, *C*) Proliferation of control vector–transduced, STAT3C^+^, or FOXP3^+^ T cells in response to anti-CD3/anti-CD28 coated microbeads (*B*) or aAPC expressing a membrane-bound OKT3scFv plus CD58 at the indicated stimulator to T cell ratios (*C*). Mean values ± sd from triplicate cultures from 1 representative experiment are depicted. *D*) Percent values normalized for control-transduced T cells from 6 experiments. n.s., not significant; **P < 0.01; ***P < 0.001.

To assess the functional consequences of STAT3 or STAT3C ectopic expression, flow cytometrically sorted *STAT3*-transduced or *STAT3C*-transduced T cells were stimulated with anti-CD3/anti-CD28 coated microbeads at different bead to cell ratios (1:1 or 1:4). Under these conditions, *STAT3*-transduced T cells showed similar proliferative capacity as control-transduced T cells. In contrast, overexpression of constitutively active *STAT3C* significantly reduced the proliferative response of transgenic T cells following both strong and weak anti-CD3/anti-CD28 mediated stimulation (*P* < 0.01 and *P* < 0.001, respectively; **Fig. 2*A***). Hyporesponsiveness of STAT3C^+^ T cells was also compared with FOXP3^+^ T cells, which are highly anergic even in response to strong stimulatory signals (25–27). Accordingly, FOXP3^+^ T cells showed >90% reduction of proliferation compared with control-transduced T cells after stimulation with anti-CD3/anti-CD28 coated microbeads (bead to cell ratio 1:1; Fig. 2*B*, *D*). Similar results were obtained on stimulation of transduced T cells with mouse thymoma BW5147 cells expressing a membrane-bound OKT3scFv and CD58 as costimulatory molecule (BW 3/2 CD58). BW 3/2 CD58 cells served as aAPCs to deliver a qualitatively different costimulatory signal than anti-CD28 (Fig. 2*C*, *D*). Overall, hyporesponsiveness by STAT3C^+^ T cells was statistically highly significant compared with control-transduced T cells; however, it was not as pronounced as with *FOXP3*-transduced T cells, leading to an average reduction of proliferation by 53.4 ± 23.5% (*n* = 6, *P* < 0.001) in response to anti-CD3/anti-CD28 stimulation (Fig. 2*B*, *D*) and 70.5 ± 10.4% (*n* = 5, *P* < 0.001) in response to anti-CD3/CD58 stimulation by aAPCs, respectively (Fig. 2*C*, *D*). STAT3C-mediated hyporesponsiveness was independent of STAT3C-induced IL-10 (see below), because neutralization of IL-10 by blocking antibodies did not alter proliferation of STAT3C-transgenic T cells after anti-CD3/anti-CD28 stimulation (*n* = 3, *P* < 0.001; Supplemental Fig. S1).

### STAT3C^+^ T cells display a Tr1-like cytokine secretion pattern

**Figure 3.**
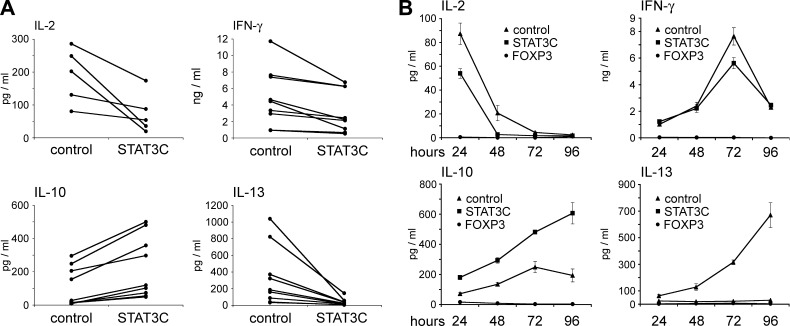
Cytokine secretion pattern of CD4^+^ T cells transduced with *STAT3C* and *FOXP3*. *A*) Control vector-transduced (left) and STAT3C^+^ (right) human CD4^+^ T cells from healthy donors (*n* ≥ 5) were stimulated with anti-CD3/anti-CD28 coated microbeads at a 1:1 ratio. Cytokine levels were assessed after 24 (IL-2) or 72 h (IFN-*γ*, IL-10, and IL-13). Each dataset is representative for mean values from triplicate cultures from individual healthy donors. *B*) Time course of cytokine secretion of STAT3C^+^ (squares), FOXP3^+^ (circles), and control-transduced (triangles) T cells from one representative donor. Mean values ± sd from triplicate values are shown. ***P < 0.001.

In a next step, we assessed whether the hyporesponsivenss observed in proliferation assays was also reflected at the level of cytokine secretion. Indeed, IL-2 secretion after 24 h was reduced by 57 ± 4% (*n* = 5, *P* < 0.001) in STAT3C^+^ T cells (**Fig. 3*A***). This effect was confirmed at the level of IL-2 transcription in Jurkat T cells expressing a luciferase reporter gene under control of the IL-2 promoter (data not shown). Secretion of IFN-*γ* (Fig. 3*A*) and TNF-*α* (data not shown) by STAT3C^+^ T cells was reduced by 35 ± 5% and 64 ± 8% (*n* = 8, *P* < 0.001), respectively, compared with control-transduced T cells, and IL-13 production was almost abrogated (reduction by 89 ± 3% compared with control-transduced T cells; *n* = 8, *P* < 0.001) after 72 h (Fig. 3*A*). Under these conditions, IL-10 production was strongly and highly significantly induced (4.1 ± 0.5-fold; *n* = 8, *P* < 0.001; Fig. 3*A*). No IL-4 and IL-17 secretion was observed (data not shown) in these experiments. Significantly, these changes in the cytokine secretion pattern of STAT3C^+^ T cells were detected over the whole course of a T cell response (Fig. 3*B*). These effects were strictly dependent on the constitutively activated form of STAT3, because wild-type *STAT3*-transduced T cells displayed a cytokine secretion profile similar to control-transduced T cells (data not shown).

### STAT3C^+^ T cells show regulatory capacity

**Figure 4.**
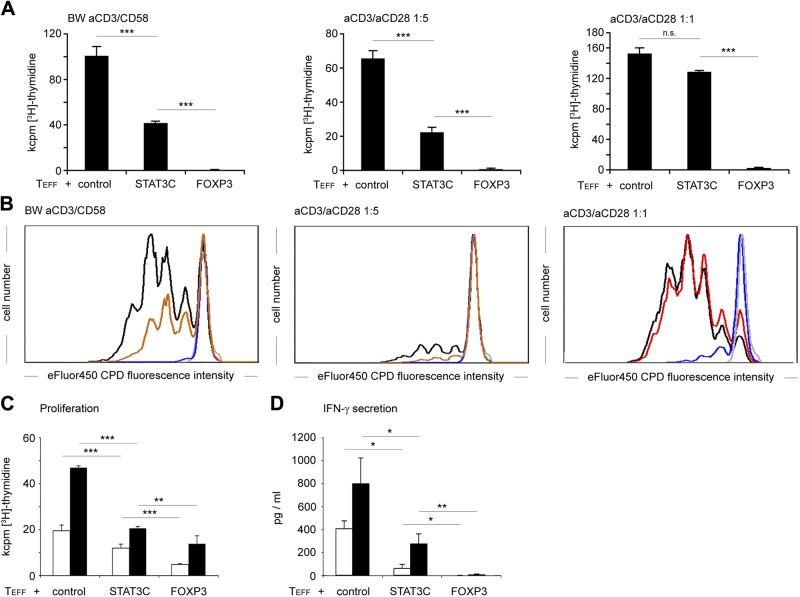
*STAT3C*-transduced CD4^+^ T cells have regulatory capacity. *A*) Purified human CD4^+^ T cells were cocultured with either STAT3C^+^, FOXP3^+^ or control vector-transduced T cells at a 1:1 ratio and stimulated with the indicated amounts of aAPC expressing a membrane-bound OKT3scFv plus CD58 or anti-CD3/anti-CD28 coated microbeads. *A*) Thymidine incorporation (72 + 16 h [^3^H]-thymidine pulse). Mean values + sd from triplicate cultures from 1 representative experiment of 4 are depicted. *B*) The graphs represent the proliferation of eFluor670 CPD-labeled CD4^+^ T cells cocultured with STAT3C^+^ (red), FOXP3^+^ (blue), or control vector-transduced (black) T cells and stimulated as indicated. One representative experiment from duplicate cultures of 4 individual donors is depicted. *C*, *D*) Purified human CD4^+^ T cells were cocultured with the indicated transgenic T cells (control vector-transduced, STAT3C^+^, or FOXP3^+^) and stimulated with HLA-mismatched immature (white) or LPS-matured (black) monocyte-derived dendritic cells at a 10:1 ratio. *C*) Proliferation after 120 + 16 h [^3^H]-thymidine pulse. *D*) IFN-*γ* secretion after 120-h coculture. Mean values ± sd from triplicate values from 1 representative donor of 4 are shown. n.s. not significant; *P < 0.05; **P < 0.01; ***P < 0.001.

Given that the cytokine and granzyme expression profile of STAT3C^+^ T cells bears resemblance to Tr1 cells, and stimulation of T cells *via* the CD58/CD2 axis enforces a Tr1 phenotype (35, 43), we hypothesized that STAT3C^+^ T cells might also exert regulatory function in this context. Indeed, STAT3C^+^ T cells activated *via* the CD58/CD2 axis by BW 3/2 CD58 aAPCs significantly suppressed T_EFF_ proliferation (reduction by 68.7 ± 10.6%, *n* = 4, *P* < 0.001; **Fig. 4*A*, *B***). Consistently, similar suppression was evident following anti-CD3/anti-CD28 mediated stimulation (bead:cell = 1:5), which still resulted in robust proliferation, *i.e.,* 40–70 kcpm. Under these conditions, STAT3C^+^ T cells significantly suppressed the proliferation of effector T cells by 65.9 ± 2.6% (*n* = 4, *P* < 0.001), whereas FOXP3^+^ T cells completely abrogated effector T cell proliferation (99.8 ± 0.1%, *n* = 4, *P* < 0,001; Fig. 4*A*, *B*). However, this suppression was overcome in response to a strong anti-CD3/anti-CD28 mediated signal (bead:cell = 1:1) where STAT3C^+^ T cells were almost unable to suppress the proliferation of cocultured CD4^+^ effector T cells (Fig. 4*A*, *B*), whereas FOXP3^+^ T cells potently suppressed effector T cell proliferation (98.6 ± 0.5%, *n* = 4, *P* < 0.001; Fig. 4*A*, *B*). To assess whether STAT3C^+^ T cells are also able to inhibit alloreactive T cell responses, we performed allogeneic mixed leukocyte reactions using immature and LPS-matured monocyte-derived dendritic cells from HLA-mismatched donors as stimulator cells. We found that coculture with STAT3C^+^ T cells significantly reduced T_EFF_ proliferation compared with cocultures with control-transduced T cells. Overall, proliferation in cocultures with STAT3C^+^ T cells was reduced by 34.2 ± 13.1% (*n* = 4; *P* < 0.001) in response to stimulation with immature dendritic cells and by 38.8 ± 16.8% (*n* = 4; *P* < 0.001) in response to LPS-matured dendritic cells (Fig. 4*C*). Under these stimulatory conditions, FOXP3-transgenic T cells showed a stronger suppressive capacity with a reduction of proliferation by 74.5 ± 10.6% (*n* = 4; *P* < 0.001) for immature DC and 70.7 ± 9.2% (n = 4; *P* < 0.001) for LPS-matured DC (Fig. 4*C*). This suppressive capacity of STAT3C^+^ T cells was also mirrored by significantly decreased IFN-*γ* levels on coculture after stimulation with both immature mdDCs (52.9 ± 33.4%; *n* = 4; *P* < 0.05) and LPS-matured mdDCs (53.1 ± 28.3%; *n* = 4; *P* < 0.05; Fig. 4*D*).

### STAT3C^+^ T cells display increased expression of granzyme B

**Figure 5.**
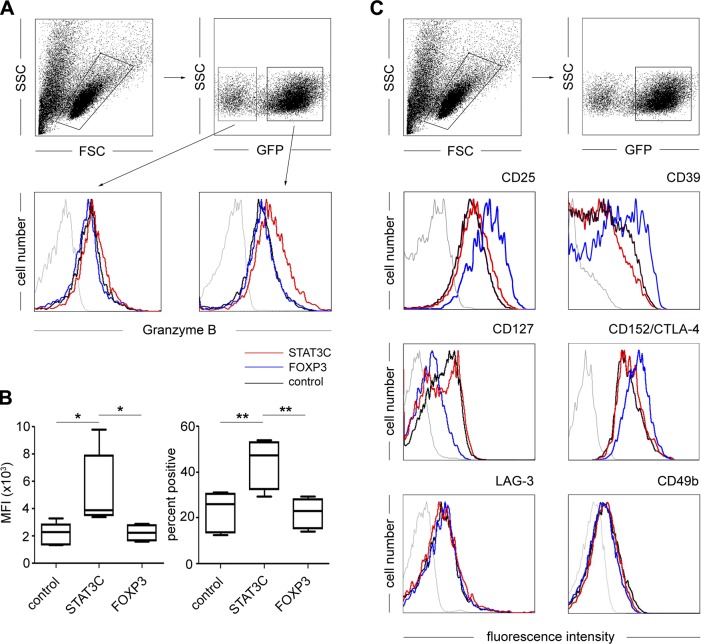
*STAT3C*-transduced T cells express an effector phenotype. Primary human CD4^+^ T cells were retrovirally transduced either with human *STAT3C* (red), *FOXP3* (blue), or an empty control vector (black). Transduced (GFP^+^) T cells were analyzed for (*A*) intracellular expression of granzyme B. Gating strategies are shown in the upper panels. Nontransduced (left) and transduced T cells (right) are depicted. *B*) Intracellular expression of granzyme B. Left panel shows box plots of mean fluorescence intensity; right panel shows box plots of percent positive cells of CD4^+^ T cells transduced with the indicated transgenes (resting phase) and stained for intracellular granzyme B expression. Data are representative of 5 healthy donors. *P < 0.05; **P < 0.01. *C*) Staining for surface expression of the regulatory T cell markers CD25, CD39, CD127, LAG3, and CD49b and intracellular expression of CD152/CTLA-4. One representative experiment of 5 is shown. Thin gray lines represent staining with isotype-matched control mAb.

Because ectopic expression of STAT3C dramatically altered the functional response of CD4^+^ T cells, we also assessed its influence on cellular phenotype. Intracellular expression of granzyme B, which has been implied as an effector molecule of Tr1 cells (44, 45), was significantly up-regulated in *STAT3C*-transgenic T cells compared with both control-transduced and *FOXP3*-transgenic T cells (mean fluorescence intensity: control, 2157 ± 727; STAT3C, 5357 ± 2422; FOXP3, 2231 ± 530; *n* = 5, *P* < 0.05; percentage of highly positive cells: control, 22.8 ± 7.9%; STAT3C, 43.9 ± 9.7%; FOXP3, 22.1 ± 5.9%; *n* = 5, *P* < 0.01; Fig. 5*A*, right, and *B*). This up-regulation was highly specific, because GFP-negative, nontransduced T cells within the same STAT3C bulk transduction experiments did not show elevated granzyme B expression levels (**Fig. 5*A***, left). Under no conditions could the expression of granzyme A be detected (data not shown). Apart from these phenotypic changes, STAT3C^+^ T cells expressed a T_EFF_ phenotype similar to control-transduced T cells, which was marked by low expression of CD25, CD39, and CTLA-4 and high expression levels of CD127. This was in contrast to *FOXP3*-transduced T cells, which displayed the nTreg-specific CD25^high^CD39^high^CTLA-4^high^CD127^low^ phenotype (Fig. 5*C*) as specified recently (27). Similarly, assessment of the Tr1 markers LAG-3 and CD49b, as well as Tr1-associated molecules CD226, A2A-R, 4-1BBL, and CD86 (22), showed no difference in the expression among the 3 groups (Fig. 5*C* and Supplemental Fig. S2).

### Suppression by STAT3C^+^ T cells is a granzyme B-dependent process

**Figure 6.**
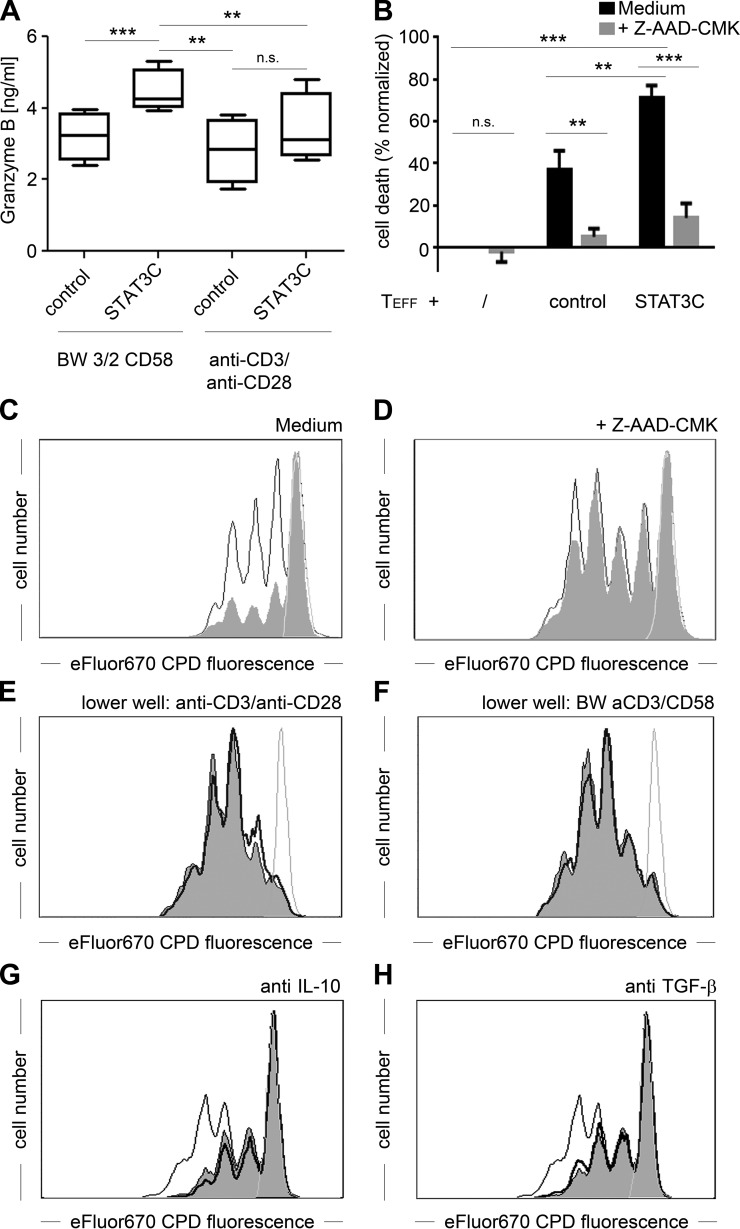
The regulatory capacity of STAT3C^+^ T cells is a granzyme B-dependent process. *A*) Release of granzyme B from control vector transduced or STAT3C-transduced T cells 6 h after stimulation with BW 3/2 CD58 aAPC (left) or anti-CD3/anti-CD28 coated microbeads (right). Cumulative data from triplicate values from 4 individual donors are depicted. *B*) Relative cell death of T_EFF_ induced by control vector- or STAT3C-transduced T cells in the presence or absence of 30 *µ*M Z-AAD-CMK as determined by combined annexin V/propidium iodide staining. *C*, *D*) CPD-labeled CD4^+^ T cells were cocultured with control vector-transduced T cells (thin black line) or STAT3C^+^ T cells (gray filled area) in the absence (*C*; medium) or presence (*D*) of 30 *µ*M Z-AAD-CMK. Nonstimulated cells are depicted as a fine gray line. One representative experiment from 4 individual donors is depicted. *E*, *F*) Transwell assay: CPD-labeled CD4^+^ T cells were stimulated with BW 3/2 CD58 aAPC in the upper well of a transwell plate (3-*µ*m pore size). Control-transduced (bold black line) or STAT3C^+^ T cells (gray filled area) were stimulated in the lower well with either (*E*) anti-CD3/anti-CD28 coated microbeads or (*F*) BW 3/2 CD58 aAPC. Nonstimulated cells are depicted as a fine gray line. *G*, *H*) CPD-labeled CD4^+^ T cells were cocultured with control-transduced T cells (fine black line) or STAT3C^+^ T cells in the presence of 20 *µ*g/ml control antibody (gray filled area) or blocking antibody (bold black line) against (*G*) IL-10 or (*H*) TGF-*β*. One representative experiment of 3 is depicted.

In accordance with the phenotypic observations, STAT3C^+^ T cells also showed significantly increased secretion of granzyme B following costimulation *via* the CD58/CD2 axis with BW 3/2 CD58 aAPCs compared with control-transduced cells (**Fig. 6*A***; 4455 ± 603 *vs*. 3220 ± 664 pg/ml; *n* = 4; *P* < 0.001). A similar tendency was found in response to anti-CD3/anti-CD28 mediated stimulation, which did, however, not reach statistical significance (3449 ± 1073 vs. 2895 ± 1020 pg/ml; *n* = 4; *P* = 0.17). To test whether this increase in granzyme B secretion was also involved in the mechanism of suppression by STAT3C^+^ T cells, we performed coculture experiments in the presence or absence of the granzyme B inhibitor Z-AAD-CMK (final concentration, 30 *µ*M). Viability of T_EFF_ was determined by combined annexin V and propidium iodide staining 72 h after activation with BW 3/2 CD58 cells. These experiments clearly demonstrated that coculture with STAT3C^+^ T cells led to massive induction of cell death in T_EFF_ (70.5 ± 5.7%, *P* < 0.001, *n* = 6), which could be almost completely overcome by the addition of the specific granzyme B inhibitor Z-AAD-CMK (14.5 ± 6.7%; Fig. 6*B* and Supplemental Fig. S3) (36). This was in contrast to coculture of T_EFF_ with control vector-transduced T cells, where induction of cell death was significantly lower (36.9 ± 8.9%; *P* < 0.01, *n* = 6).

Additionally, Z-AAD-CMK completely abrogated the suppression of T_EFF_ proliferation by STAT3C^+^ T cells (division index, 2.10 ± 0.07 for cocultures with STAT3C^+^ T cells *vs*. 2.19 ± 0.13 for control-transduced T cells; *n* = 4, *P* = 0.09), which in the absence of the inhibitor amounted to 40.0 ± 4.7% (division index, 1.50 ± 0.07 *vs*. 0.90 ± 0.07; *n* = 4, *P* < 0.001; Fig. 6*B*). Remarkably, addition of Z-AAD-CMK also led to an increase of T_EFF_ proliferation by 29.0 ± 3.3% in cocultures with control-transduced T cells. This is compatible with the constitutively up-regulated granzyme B levels observed in these cells. The finding that suppression by STAT3C^+^ T cells is mediated by granzyme B is in line with transwell experiments showing that their suppressive capacity is contact dependent (Fig. 6*C*, *D*), which is a classic feature of granzyme B-mediated cytotoxicity (46). We also assessed the contribution of IL-10 and TGF-*β* to the suppressive capacity of STAT3C^+^ T cells, because these cytokines have been reported to be key mediators of Tr1 cell regulatory function (14, 45, 47–49). For that purpose, cocultures of CD4^+^ T_EFF_ with *STAT3C*-transduced or control-transduced T cells were stimulated with BW 3/2 CD58 aAPCs and supplemented with either a nonbinding control mAb or blocking antibodies to IL-10 or TGF-*β*. Neutralization of neither IL-10 (Fig. 6*E*) nor TGF-*β* (Fig. 6*F*) led to the abrogation of the suppressive capacity of STAT3C^+^ T cells, indicating that these cytokines do not constitute the main T cell regulatory mechanisms of STAT3C^+^ T cells.

### Tr1 cells from peripheral blood show increased STAT3 phosphorylation on activation

**Figure 7.**
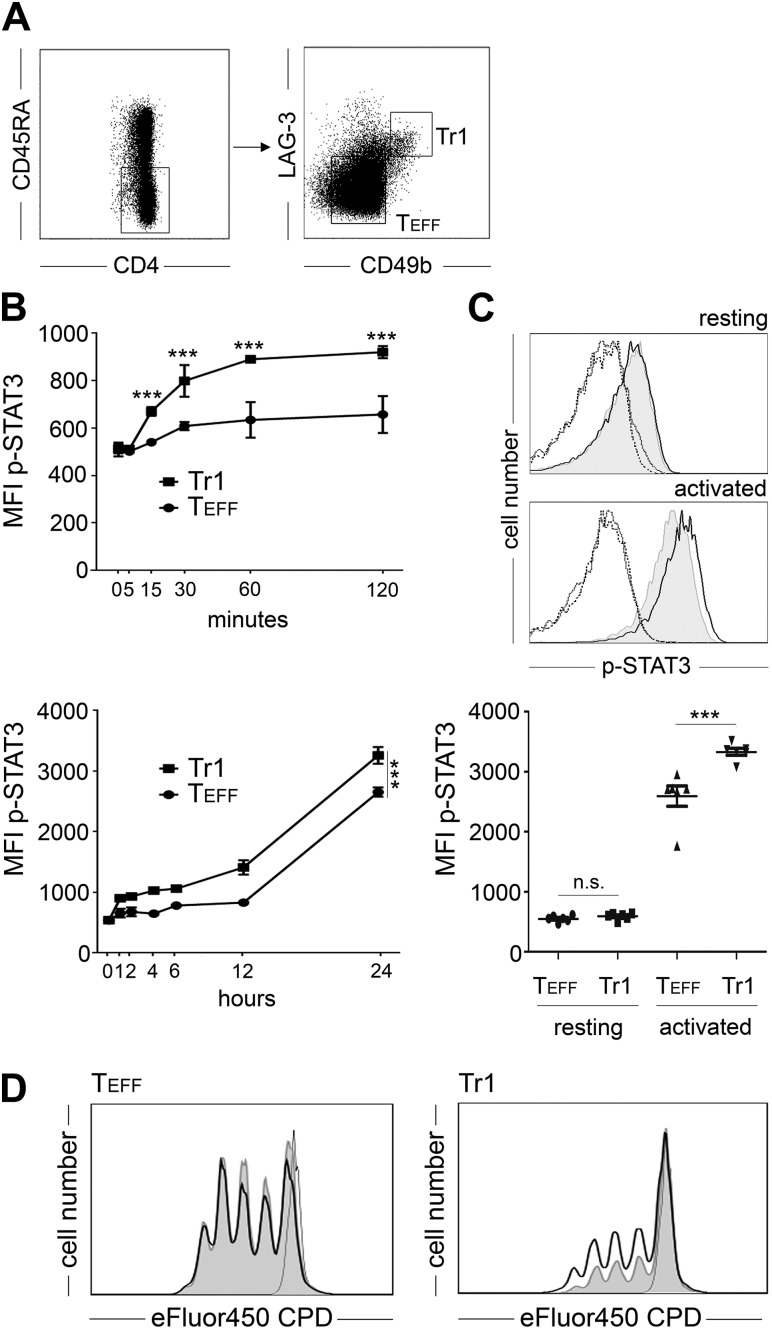
Activation-induced hyperphosphorylation and functional potential of STAT3 in Tr1 cells. *A*) Magnetic-activated cell-sorted CD4^+^ T cells were stained for surface expression of CD4, CD45RA, and the Tr1 markers CD49b, and LAG-3 and CD4^+^CD45RA^−^ cells were further FACS sorted into T_EFF_ and Tr1 cells according to the indicated gates. *B*) FACS-sorted Tr1 cells (black squares) or T_EFF_ (black circles) was analyzed for STAT3 phosphorylation, indicated as mean fluorescence intensity (MFI p-STAT3, mean ± sd, *n* = 3) after anti-CD3/anti-CD28 mediated stimulation at the indicated early (upper graph) and late (lower graph) time points. *C*) FACS-sorted Tr1 cells (bold black line) or T_EFF_ (gray histogram) was analyzed for phosphorylation of STAT3 directly after sorting (resting, upper panel) or 24 h after anti-CD3/anti-CD28 mediated stimulation (activated, lower panel). Isotype matched antibodies (dotted line for Tr1, dashed line for T_EFF_) served as controls. Data from 1 representative donor are depicted. Cumulative data for STAT3 phosphorylation from 6 individual donors are depicted in the lower diagram. *D*) eFluor450 CPD-labeled CD4^+^ T cells were sorted according to *A* and then stimulated with anti-CD3/anti-CD28 coated microbeads for 96 h in the absence (gray histogram) or presence (bold black line) of 100 *µ*M STATTIC. Unstimulated cells (fine black line) served as controls. Left, LAG3^-^CD49b^−^ T_EFF_; right, LAG3^+^CD49b^+^ Tr1 cells. Data from 1 representative experiment are depicted (*n* = 4). n.s., not significant; ***P < 0.001.

Overexpression of STAT3C confers combined functional properties, such as hyporesponsiveness and secretion of IL-10 and granzyme B, which are hallmarks of Tr1 cells. Accordingly, we assessed whether activation, *i.e.,* phosphorylation of STAT3, was also apparent in *bona fide* Tr1 cells isolated from PB. For that purpose, we relied on the recently described CD4^+^CD45RA^−^CD49b^+^LAG-3^+^ phenotype (22) to purify Tr1 cells from PB of healthy individuals (**Fig. 7*A***). Using phosphoprotein-specific flow cytometry, we compared STAT3 phosphorylation in FACS-sorted Tr1 cells with CD4^+^CD45RA^−^CD49b^−^LAG-3^−^ T_EFF_ of unrelated healthy individuals. Directly after isolation from PB no difference in STAT3 phosphorylation was observed between Tr1 and T_EFF_ (mean fluorescence intensity, 591 ± 53 *vs*. 550 ± 51; *n* = 6, *P* = 0.32; Fig 7*B*, *C*). After activation, a marked up-regulation of STAT3 phosphorylation was observed. In fact, as early as 15 min after activation, Tr1 cells showed significantly higher levels of STAT3 phosphorylation than T_EFF_ (Fig. 7*B*), which was consistently found during the course of early cellular activation. Of note, the initial increase in STAT3 phosphorylation underwent a further boost between 12 and 24 h, which is consistent with a bimodal course of STAT3 activation. After 24 h, a pronounced difference in STAT3 phosphorylation was evident between Tr1 and T_EFF_ specimens of all donors (mean fluorescence intensity, 3330 ± 193 *vs.* 2591 ± 417; *n* = 6; *P* < 0.001; Fig. 7*C*). To confirm the functional relevance of this observation, we stimulated FACS-sorted Tr1 and T_EFF_ cells in the presence or absence of STATTIC, which is a small-molecule inhibitor of STAT3 activation and dimerization (37). Addition of STATTIC partially reversed hyporesponsiveness of Tr1 cells (division index, 0.46 ± 0.11 *vs*. 0.89 ± 0.04; *n* = 3, *P* < 0.01; Fig. 7*D*). As expected, CD49b^+^LAG-3^+^ Tr1 cells showed significantly decreased proliferation in comparison to CD49b^−^LAG-3^−^ T_EFF_ (division index, 0.46 ± 0.11 *vs*. 2.27 ± 0.25; *n* = 3, *P* < 0.001). Although, CD49b^−^LAG-3^−^ T_EFF_ also presented with an activation-dependent phosphorylation of STAT3, their proliferation was unaffected by the blockade of STAT3 activation (division index, 2.27 ± 0.25 *vs*. 2.30 ± 0.18; *n* = 3, not significant).

## Discussion

To analyze the role of STAT3 in CD4^+^ T cells, we resorted to ectopic expression of a constitutively active form of STAT3, *i.e.,* STAT3C. A similar approach has previously been used to address STAT3 function in monocytes and dendritic cells (1–3, 5). Activation of STAT3 in CD4^+^ T cells dramatically altered their functional response to qualitatively and quantitatively different stimuli and induced a specific tolerogenic program. This included hyporesponsiveness to T cell receptor–mediated stimulation, the adoption of a Tr1-like cytokine and granzyme B secretion profile, and the acquisition of suppressive capacity. Furthermore, we demonstrate that suppression by STAT3C^+^ T cells is granzyme B and contact dependent but IL-10 and TGF-*β* independent. Although we thus observed a dramatic change in the functional features of STAT3C transgenic T cells, this was not reflected in their surface phenotype. In fact, phenotypic traits of regulatory T cells such as expression patterns of the nTreg markers CD25, CD39, CD127, CTLA-4, and FOXP3 and the Tr1 markers LAG-3, CD49b, CD226, A2A-R, 4-1BBL, and CD86 were unaffected, Finally, we provide the first evidence that activation-induced hyperphosphorylation of STAT3 is a typical feature of PB Tr1 cells, which are involved in the maintenance of their hypoproliferative state.

The tolerogenic effects of STAT3C were compared with those induced by ectopic expression of FOXP3, which mimics the phenotype and function of CD4^+^CD25^+^ nTregs to a great extent (25–27). These comparative analyses indeed revealed that the STAT3C-mediated tolerogenic program is distinct from the FOXP3-mediated, nTreg-like tolerogenic program. In natural Tregs or *FOXP3*-transgenic Tregs (25–27), FOXP3 completely abrogates proliferation and cytokine production including IL-10. In contrast, STAT3C^+^ T cells retain some proliferative capacity, however, at a significantly reduced level, compared with control-transgenic cells. Moreover, they display a distinct cytokine profile, which includes a >4-fold up-regulation of IL-10 paralleled by the down-regulation of IFN-*γ*, TNF-*α*, IL-2, and IL-13. This cytokine profile strongly resembles the IL-10^high^IFN-*γ*^+^IL-2^low^IL4^−^IL13^−^ phenotype, which has been described as a hallmark of Tr1 cells (14, 21). Our results comply with recent data that the activation of STAT3 induces the modulation of cytokine production in CD4^+^ T cells, in particular the induction of IL-10 production (19, 20) and the down-regulation of IL-2 production (50). Furthermore, this cytokine secretion profile is combined with significantly increased intracellular expression and secretion levels of granzyme B, a feature that has also been attributed to *in vitro*-induced Tr1 cells (44, 45). In this respect, our observations derived from ectopic expression studies are concordant with recent reports describing STAT3 as a crucial signaling molecule in the regulation of IL-10 expression (51) and the induction of Tr1 cells in response to IL-27 stimulation (3, 16–19).

Mechanistically, the effects observed in STAT3C^+^ T cells cannot be reduced to the mere induction of IL-10 secretion, because neutralization of IL-10 was not able to abrogate their hypoproliferative state. Thus, it seems that the observed functional features of STAT3C-transgenic T cells are directly governed by the transcription factor activity of STAT3, and IL-10 induction represents 1 feature of the STAT3 transcription signature in T cells.

However, in the present study, STAT3C transgenic T cells did not produce IL-17, indicating that the sole activation of STAT3 is not *per se* a Th17-polarizing signal in human T cells. This is in marked contrast to a multitude of reports studying the function of murine T cells, which have linked the IL-6-STAT3 axis to Th17 induction and subsequently to Th17-mediated pathologies such as inflammatory bowel disease (7–9). However, in some of these studies, IFN-*γ* was described to potently inhibit Th17 cytokine production in CD4^+^ T cells (8). Along those lines, it seems conceivable that the residual production of IFN-*γ* by STAT3C^+^ T cells as observed in this study (Fig. 3*A, B*) might be sufficient to suppress IL-17 production in a negative feedback loop. The makeup of the STAT3C construct (40), which most likely does not allow association with other STAT members, might also influence the further polarization of *STAT3C*-transduced cells. In this respect, the results observed in this study may certainly reflect the effects of STAT3 homodimers; they might, however, miss concomitant activation steps because of the engagement of other members of the STAT family and subsequent STAT3 heterodimerization effects.

Given that overexpression of STAT3C in CD4^+^ T cells reconstituted several functional features of Tr1 cells, we hypothesized that these cells might also feature regulatory capacity. Tr1 cells use multiple mechanisms to exert their regulatory capacity, including the secretion of inhibitory cytokines such as IL-10 and TGF-*β*, as well as killing of target cells *via* release of perforin and granzyme B (44, 45). These latter mechanisms have been further defined for the killing of myeloid APCs and are dependent on interaction of Tr1 cell-expressed CD2, CD226, and LFA-1 with their respective ligands on APCs (44). In this study, clear-cut suppressive effects of *STAT3C*-transduced T cells could only be observed when the costimulatory signal was provided *via* the CD58/CD2 axis or at suboptimal levels of CD28 costimulation. This is of special interest, because CD58-CD2 interactions, although among of the most potent T cell costimulatory pathways (30, 52, 53), seem to serve an important role in Tr1-mediated tolerance. First reports by Wakkach *et al*. indicated that costimulation *via* CD2 but not *via* CD28 induced a Tr1-like cytokine profile marked by secretion of high levels of IL-10 and decreased levels of IL-2 and IFN-*γ* (35), a feature that could be reproduced by us previously by applying a virus-like particle-based stimulatory platform (43). Along those lines, direct suppression of T cell activation by IL-10 is mainly achieved when T cells are costimulated *via* CD2 but not *via* CD28 (54). Similarly, suppressive effects of Tr1 cells *via* granzyme B and perforin are dependent on CD2-CD58 interaction (44). In our system, both control-transduced and STAT3C-transduced T cells express and secrete robust amounts of granzyme B. This finding is most likely explained by the preactivation needed for transduction, which is in line with reports that preactivation of CD4^+^ T cells leads to strong up-regulation of granzyme B (45). However, transduction with STAT3C further enhanced granzyme B expression above the activation-induced levels, indicating that STAT3 might be involved in the up-regulation of granzyme B expression in CD4^+^ T cells. Elevated expression of granzyme B in STAT3C^+^ T cells was also reflected by increased granzyme B secretion levels following activation, an effect that was more strongly favored by CD58/CD2 costimulation than by CD28 costimulation. In line with the observations described by Magnani *et al*. (44), this may indicate that costimulation *via* the CD58/CD2 axis is a prerequisite for the secretion of granzyme B from tolerogenic CD4^+^ T cells. Indeed, the mechanism of action constituted by STAT3C^+^ T cells was confirmed to be caused by granzyme B-dependent induction of T_EFF_ cell death. Along those lines, we observed increased levels of cell death on coculture of T_EFF_ with STAT3C^+^ T cells, which could be overcome by the addition of the specific granzyme B inhibitor Z-AAD-CMK (36), a widely used peptide chloromethyl ketone with selective blocking activity for granzyme B but not granzyme A or K (36, 55–57). Consequently, addition of Z-AAD-CMK also significantly improved T_EFF_ proliferation when cocultured with STAT3C^+^ T cells. Taken together, these findings provide a plausible explanation that suppression is granzyme B dependent and therefore mainly observed following costimulation of T_EFF_ *via* the CD58/CD2 axis.

In a final step, we aimed to assess whether the overexpression system applied in this study is of physiologic relevance, *i.e.,* whether activation of STAT3 might be part of the transcriptional signature of human PB Tr1 cells. Although a possible link between IL-10 signaling, STAT3 phosphorylation, and the induction of Tr1 cells has been suggested by several studies, no formal proof for this hypothesis has been provided as of today. Recently, Gagliani *et al.* identified the combined expression of CD49b and LAG3 as unique markers of CD4^+^CD45RA^−^ PB Tr1 cells (22). On the basis of these observations, we compared STAT3 phosphorylation in Tr1 cells with effector T cells from PB. Although basal levels of STAT3 phosphorylation were similar in both populations, Tr1 cells showed more pronounced up-regulation of STAT3 phosphorylation following activation. Detailed time course analyses revealed a quick first wave of neo-phosphorylation of STAT3 in Tr1 cells, which was almost undetectable in T_EFF_ cells. Between 12 and 24 h after activation, a second wave of STAT3 phosphorylation became evident, which was also regularly detectable in T_EFF_, although at a significantly lower magnitude. It is tempting to speculate that a putative feedback mechanism driven by IL-10 secretion could account for this effect. Consistently, pharmacologic blockade of STAT3 activation with the selective inhibitor STATTIC (37) partially reversed hypoproliferation of Tr1 cells, indicating that STAT3 hyperphosphorylation might be involved in the functional features of Tr1 cells. STATTIC has a high potency at 37°C and does not significantly inhibit other dimeric transcription factors such as c-myc/Max, Jun/Jun, and STAT1 or other important signaling molecules such as Src, JAK1, JAK2, Akt, JNK, and ERK1/2 (37). To our knowledge, these data provide the first evidence for an important role of STAT3 in Tr1 cells isolated from PB, because most studies have thus far relied on *in vitro*-induced Tr1 cells.

In concert with the data obtained in STAT3C overexpression experiments, it seems conceivable that Tr1 cells maintain increased STAT3 phosphorylation following activation to stabilize hyporesponsiveness and secretion of IL-10 and granzyme B, as well as down-regulation of other effector cytokines. Although many functional and phenotypic traits of Tr1 cells can be recapitulated by ectopic expression of STAT3C, surface expression of typical markers designating Tr1 cells, such as CD49b, LAG-3, CD226, A2A-R, 4-1BBL, and CD86, remain unaffected. Several reasons might account for this discrepancy.

One possiblility is that the manifestation of phenotypic traits of PB Tr1 cells are independent of STAT3 activation or require additional signals. Our findings that resting Tr1 cells do not show increased levels of phosphorylation of STAT3 support this hypothesis. Taken together with our results that blockade of STAT3 was not fully sufficient to revert hypoproliferation by Tr1 cells, it seems highly likely that the Tr1 transcriptional signature consists of >1 transcription factor necessary for the full manifestation of their phenotype and function. Along those lines, it has been shown that p38 signaling is a crucial cofactor for the induction of LAG-3 expression (58). Furthermore, transcription factors, such as c-Maf (59) and the aryl hydrocarbon receptor (60), might represent additional elements. It will be interesting to assess individual contributions of these factors in future studies by applying multicistronic overexpression approaches (27, 61). The findings here also might have potential practical implications, providing a general strategy of how to engineer Tr1-like cells for therapeutic application by genetic manipulation, similar to the recently described IL-10 overexpression approaches from Andolfi *et al.* (62).

In conclusion, it seems conceivable that sustained phosphorylation of STAT3 in human T cells, potentially maintained by elevated levels of IL-10 and IL-27, induces a tolerogenic program. This includes the adoption of hyporesponsiveness, an anti-inflammatory cytokine secretion pattern, as well as increased expression and secretion of granzyme B, which executes direct suppressive function on bystander effector T cells in a contact-dependent manner. This mechanism is dependent on costimulation *via* the CD58-CD2 axis. Our observations are further substantiated by the finding that activated PB Tr1 cells reveal increased STAT3 phosphorylation that helps to maintain their hypoproliferative state, indicating that the STAT3-mediated tolerogenic program among possible other cell types described herein might be involved in the tolerogenic functions of *bona fide* PB Tr1 cells.

## Supplementary Material

Supplemental Data

## Abbreviations

aAPC: artificial antigen-presenting cell
CD: cluster of differentiation
CTLA: cytotoxic T-lymphocyte antigen
FACS: fluorescence-activated cell sorting
FOXP3: forkhead box protein 3
GFP: green fluorescent protein
IRES: internal ribosomal entry site
LAG-3: lymphocyte activation gene-3
mdDC: monocyte-derived dendritic cells
nTreg: naturally occurring regulatory T cells
PB: peripheral blood
scFv: single-chain variable fragment
STAT: signal transducer and activator of transcription
T_EFF_: effector T cell
Th: T-helper cell
Treg: regulatory T cells
Tr1: type 1 regulatory T cells
Z-AAD-CMK: benzyloxycarbonyl-ala-ala-asp(OMe) chloromethyl ketone

## References

[1] Cheng F., Wang H. W., Cuenca A., Huang M., Ghansah T., Brayer J., Kerr W. G., Takeda K., Akira S., Schoenberger S. P., Yu H., Jove R., Sotomayor E. M. (2003) A critical role for Stat3 signaling in immune tolerance. Immunity 19, 425–436 14499117 10.1016/s1074-7613(03)00232-2

[2] Herrmann A., Kortylewski M., Kujawski M., Zhang C., Reckamp K., Armstrong B., Wang L., Kowolik C., Deng J., Figlin R., Yu H. (2010) Targeting Stat3 in the myeloid compartment drastically improves the in vivo antitumor functions of adoptively transferred T cells. Cancer Res. 70, 7455–7464 20841481 10.1158/0008-5472.CAN-10-0736 PMC3058618

[3] Wang T., Niu G., Kortylewski M., Burdelya L., Shain K., Zhang S., Bhattacharya R., Gabrilovich D., Heller R., Coppola D., Dalton W. Jove R. Pardoll D. Yu H. (2004) Regulation of the innate and adaptive immune responses by Stat-3 signaling in tumor cells. Nat. Med. 10, 48–54 14702634 10.1038/nm976

[4] Matta B. M., Raimondi G., Rosborough B. R., Sumpter T. L., Thomson A. W. (2012) IL-27 production and STAT3-dependent upregulation of B7-H1 mediate immune regulatory functions of liver plasmacytoid dendritic cells. J. Immunol. 188, 5227–5237 22508931 10.4049/jimmunol.1103382 PMC3564546

[5] Williams L. M., Sarma U., Willets K., Smallie T., Brennan F., Foxwell B. M. (2007) Expression of constitutively active STAT3 can replicate the cytokine-suppressive activity of interleukin-10 in human primary macrophages. J. Biol. Chem. 282, 6965–6975 17194701 10.1074/jbc.M609101200

[6] Kaebisch R., Mejías-Luque R., Prinz C., Gerhard M. (2014) Helicobacter pylori cytotoxin-associated gene A impairs human dendritic cell maturation and function through IL-10-mediated activation of STAT3. J. Immunol. 192, 316–323 24293633 10.4049/jimmunol.1302476

[7] Mathur A. N., Chang H. C., Zisoulis D. G., Stritesky G. L., Yu Q., O’Malley J. T., Kapur R., Levy D. E., Kansas G. S., Kaplan M. H. (2007) Stat3 and Stat4 direct development of IL-17-secreting Th cells. J. Immunol. 178, 4901–4907 17404271 10.4049/jimmunol.178.8.4901

[8] Yang X. O., Panopoulos A. D., Nurieva R., Chang S. H., Wang D., Watowich S. S., Dong C. (2007) STAT3 regulates cytokine-mediated generation of inflammatory helper T cells. J. Biol. Chem. 282, 9358–9363 17277312 10.1074/jbc.C600321200

[9] Zhou L., Ivanov I. I., Spolski R., Min R., Shenderov K., Egawa T., Levy D. E., Leonard W. J., Littman D. R. (2007) IL-6 programs T(H)-17 cell differentiation by promoting sequential engagement of the IL-21 and IL-23 pathways. Nat. Immunol. 8, 967–974 17581537 10.1038/ni1488

[10] Kwon M. J., Ma J., Ding Y., Wang R., Sun Z. (2012) Protein kinase C-θ promotes Th17 differentiation *via* upregulation of Stat3. J. Immunol. 188, 5887–5897 22586032 10.4049/jimmunol.1102941 PMC3484892

[11] Chaudhry A., Rudra D., Treuting P., Samstein R. M., Liang Y., Kas A., Rudensky A. Y. (2009) CD4+ regulatory T cells control TH17 responses in a Stat3-dependent manner. Science 326, 986–991 19797626 10.1126/science.1172702 PMC4408196

[12] Chaudhry A., Samstein R. M., Treuting P., Liang Y., Pils M. C., Heinrich J. M., Jack R. S., Wunderlich F. T., Brüning J. C., Müller W., Rudensky A. Y. (2011) Interleukin-10 signaling in regulatory T cells is required for suppression of Th17 cell-mediated inflammation. Immunity 34, 566–578 21511185 10.1016/j.immuni.2011.03.018 PMC3088485

[13] Pallandre J. R., Brillard E., Créhange G., Radlovic A., Remy-Martin J. P., Saas P., Rohrlich P. S., Pivot X., Ling X., Tiberghien P., Borg C. (2007) Role of STAT3 in CD4+CD25+FOXP3+ regulatory lymphocyte generation: implications in graft-*versus*-host disease and antitumor immunity. J. Immunol. 179, 7593–7604 18025205 10.4049/jimmunol.179.11.7593

[14] Groux H., O’Garra A., Bigler M., Rouleau M., Antonenko S., de Vries J. E., Roncarolo M. G. (1997) A CD4+ T-cell subset inhibits antigen-specific T-cell responses and prevents colitis. Nature 389, 737–742 9338786 10.1038/39614

[15] Levings M. K., Gregori S., Tresoldi E., Cazzaniga S., Bonini C., Roncarolo M. G. (2005) Differentiation of Tr1 cells by immature dendritic cells requires IL-10 but not CD25+CD4+ Tr cells. Blood 105, 1162–1169 15479730 10.1182/blood-2004-03-1211

[16] Levings M. K., Sangregorio R., Galbiati F., Squadrone S., de Waal Malefyt R., Roncarolo M. G. (2001) IFN-alpha and IL-10 induce the differentiation of human type 1 T regulatory cells. J. Immunol. 166, 5530–5539 11313392 10.4049/jimmunol.166.9.5530

[17] Awasthi A., Carrier Y., Peron J. P., Bettelli E., Kamanaka M., Flavell R. A., Kuchroo V. K., Oukka M., Weiner H. L. (2007) A dominant function for interleukin 27 in generating interleukin 10-producing anti-inflammatory T cells. Nat. Immunol. 8, 1380–1389 17994022 10.1038/ni1541

[18] Fitzgerald D. C., Zhang G. X., El-Behi M., Fonseca-Kelly Z., Li H., Yu S., Saris C. J., Gran B., Ciric B., Rostami A. (2007) Suppression of autoimmune inflammation of the central nervous system by interleukin 10 secreted by interleukin 27-stimulated T cells. Nat. Immunol. 8, 1372–1379 17994023 10.1038/ni1540

[19] Stumhofer J. S., Silver J. S., Laurence A., Porrett P. M., Harris T. H., Turka L. A., Ernst M., Saris C. J., O’Shea J. J., Hunter C. A. (2007) Interleukins 27 and 6 induce STAT3-mediated T cell production of interleukin 10. Nat. Immunol. 8, 1363–1371 17994025 10.1038/ni1537

[20] Wang H., Meng R., Li Z., Yang B., Liu Y., Huang F., Zhang J., Chen H., Wu C. (2011) IL-27 induces the differentiation of Tr1-like cells from human naive CD4+ T cells *via* the phosphorylation of STAT1 and STAT3. Immunol. Lett. 136, 21–28 21115047 10.1016/j.imlet.2010.11.007

[21] Gregori S., Goudy K. S., Roncarolo M. G. (2012) The cellular and molecular mechanisms of immuno-suppression by human type 1 regulatory T cells. Front. Immunol. 3, 30 22566914 10.3389/fimmu.2012.00030 PMC3342353

[22] Gagliani N., Magnani C. F., Huber S., Gianolini M. E., Pala M., Licona-Limon P., Guo B., Herbert D. R., Bulfone A., Trentini F., Di Serio C., Bacchetta R., Andreani M., Brockmann L., Gregori S., Flavell R. A., Roncarolo M. G. (2013) Coexpression of CD49b and LAG-3 identifies human and mouse T regulatory type 1 cells. Nat. Med. 19, 739–746 23624599 10.1038/nm.3179

[23] Ozcan E., Notarangelo L. D., Geha R. S. (2008) Primary immune deficiencies with aberrant IgE production. J. Allergy Clin. Immunol. 122, 1054–1062 19084106 10.1016/j.jaci.2008.10.023

[24] Haga S., Terui K., Zhang H. Q., Enosawa S., Ogawa W., Inoue H., Okuyama T., Takeda K., Akira S., Ogino T., Irani K., Ozaki M. (2003) Stat3 protects against Fas-induced liver injury by redox-dependent and -independent mechanisms. J. Clin. Invest. 112, 989–998 14523036 10.1172/JCI17970 PMC198521

[25] Aarts-Riemens T., Emmelot M. E., Verdonck L. F., Mutis T. (2008) Forced overexpression of either of the two common human Foxp3 isoforms can induce regulatory T cells from CD4(+)CD25(-) cells. Eur. J. Immunol. 38, 1381–1390 18412171 10.1002/eji.200737590

[26] Allan S. E., Alstad A. N., Merindol N., Crellin N. K., Amendola M., Bacchetta R., Naldini L., Roncarolo M. G., Soudeyns H., Levings M. K. (2008) Generation of potent and stable human CD4+ T regulatory cells by activation-independent expression of FOXP3. Mol. Ther. 16, 194–202 17984976 10.1038/sj.mt.6300341

[27] Schmetterer K. G., Haiderer D., Leb-Reichl V. M., Neunkirchner A., Jahn-Schmid B., Kung H. J., Schuch K., Steinberger P., Bohle B., Pickl W. F. (2011) Bet v 1-specific T-cell receptor/forkhead box protein 3 transgenic T cells suppress Bet v 1-specific T-cell effector function in an activation-dependent manner. J. Allergy Clin. Immunol. 127, 238–245 21211658 10.1016/j.jaci.2010.10.023

[28] Popow I., Leitner J., Grabmeier-Pfistershammer K., Majdic O., Zlabinger G. J., Kundi M., Steinberger P. (2013) A comprehensive and quantitative analysis of the major specificities in rabbit antithymocyte globulin preparations. Am. J. Transplant. 13, 3103–3113 24168235 10.1111/ajt.12514

[29] Derdak S. V., Kueng H. J., Leb V. M., Neunkirchner A., Schmetterer K. G., Bielek E., Majdic O., Knapp W., Seed B., Pickl W. F. (2006) Direct stimulation of T lymphocytes by immunosomes: virus-like particles decorated with T cell receptor/CD3 ligands plus costimulatory molecules. Proc. Natl. Acad. Sci. USA 103, 13144–13149 16924110 10.1073/pnas.0602283103 PMC1559767

[30] Leitner J., Kuschei W., Grabmeier-Pfistershammer K., Woitek R., Kriehuber E., Majdic O., Zlabinger G., Pickl W. F., Steinberger P. (2010) T cell stimulator cells, an efficient and versatile cellular system to assess the role of costimulatory ligands in the activation of human T cells. J. Immunol. Methods 362, 131–141 20858499 10.1016/j.jim.2010.09.020 PMC2975062

[31] Leb V. M., Jahn-Schmid B., Schmetterer K. G., Kueng H. J., Haiderer D., Neunkirchner A., Fischer G. F., Nissler K., Hartl A., Thalhamer J., Bohle B., Seed B., Pickl W. F. (2008) Molecular and functional analysis of the antigen receptor of Art v 1-specific helper T lymphocytes. J. Allergy Clin. Immunol. 121, 64–71 18037161 10.1016/j.jaci.2007.10.006

[32] Neunkirchner A., Leb-Reichl V. M., Schmetterer K. G., Mutschlechner S., Kueng H. J., Haiderer D., Schuch K., Wallner M., Jahn-Schmid B., Bohle B., Pickl W. F. (2011) Human TCR transgenic Bet v 1-specific Th1 cells suppress the effector function of Bet v 1-specific Th2 cells. J. Immunol. 187, 4077–4087 21908735 10.4049/jimmunol.1003220

[33] Groux H., Bigler M., de Vries J. E., Roncarolo M. G. (1998) Inhibitory and stimulatory effects of IL-10 on human CD8+ T cells. J. Immunol. 160, 3188–3193 9531274

[34] Murugaiyan G., Mittal A., Lopez-Diego R., Maier L. M., Anderson D. E., Weiner H. L. (2009) IL-27 is a key regulator of IL-10 and IL-17 production by human CD4+ T cells. J. Immunol. 183, 2435–2443 19625647 10.4049/jimmunol.0900568 PMC2904948

[35] Wakkach A., Cottrez F., Groux H. (2001) Differentiation of regulatory T cells 1 is induced by CD2 costimulation. J. Immunol. 167, 3107–3113 11544295 10.4049/jimmunol.167.6.3107

[36] Kam C. M., Hudig D., Powers J. C. (2000) Granzymes (lymphocyte serine proteases): characterization with natural and synthetic substrates and inhibitors. Biochim. Biophys. Acta 1477, 307–323 10708866 10.1016/s0167-4838(99)00282-4

[37] Schust J., Sperl B., Hollis A., Mayer T. U., Berg T. (2006) Stattic: a small-molecule inhibitor of STAT3 activation and dimerization. Chem. Biol. 13, 1235–1242 17114005 10.1016/j.chembiol.2006.09.018

[38] Asquith B., Debacq C., Florins A., Gillet N., Sanchez-Alcaraz T., Mosley A., Willems L. (2006) Quantifying lymphocyte kinetics in vivo using carboxyfluorescein diacetate succinimidyl ester (CFSE). Proc. Biol. Sci. 273, 1165–1171 16600897 10.1098/rspb.2005.3432 PMC1560268

[39] Steinberger P., Majdic O., Derdak S. V., Pfistershammer K., Kirchberger S., Klauser C., Zlabinger G., Pickl W. F., Stöckl J., Knapp W. (2004) Molecular characterization of human 4Ig-B7-H3, a member of the B7 family with four Ig-like domains. J. Immunol. 172, 2352–2359 14764704 10.4049/jimmunol.172.4.2352

[40] Bromberg J. F., Wrzeszczynska M. H., Devgan G., Zhao Y., Pestell R. G., Albanese C., Darnell J. E. Jr (1999) Stat3 as an oncogene. Cell 98, 295–303 10458605 10.1016/s0092-8674(00)81959-5

[41] Hori S., Nomura T., Sakaguchi S. (2003) Control of regulatory T cell development by the transcription factor Foxp3. Science 299, 1057–1061 12522256 10.1126/science.1079490

[42] Yagi H., Nomura T., Nakamura K., Yamazaki S., Kitawaki T., Hori S., Maeda M., Onodera M., Uchiyama T., Fujii S., Sakaguchi S. (2004) Crucial role of FOXP3 in the development and function of human CD25+CD4+ regulatory T cells. Int. Immunol. 16, 1643–1656 15466453 10.1093/intimm/dxh165

[43] Leb V. M., Jahn-Schmid B., Kueng H. J., Schmetterer K. G., Haiderer D., Neunkirchner A., Fischer G. F., Hartl A., Thalhamer J., Steinberger P., Bohle B., Seed B., Pickl W. F. (2009) Modulation of allergen-specific T-lymphocyte function by virus-like particles decorated with HLA class II molecules. J. Allergy Clin. Immunol. 124, 121–128 19500826 10.1016/j.jaci.2009.04.008

[44] Magnani C. F., Alberigo G., Bacchetta R., Serafini G., Andreani M., Roncarolo M. G., Gregori S. (2011) Killing of myeloid APCs *via* HLA class I, CD2 and CD226 defines a novel mechanism of suppression by human Tr1 cells. Eur. J. Immunol. 41, 1652–1662 21469116 10.1002/eji.201041120 PMC3116154

[45] Grossman W. J., Verbsky J. W., Tollefsen B. L., Kemper C., Atkinson J. P., Ley T. J. (2004) Differential expression of granzymes A and B in human cytotoxic lymphocyte subsets and T regulatory cells. Blood 104, 2840–2848 15238416 10.1182/blood-2004-03-0859

[46] Gondek D. C., Lu L. F., Quezada S. A., Sakaguchi S., Noelle R. J. (2005) Cutting edge: contact-mediated suppression by CD4+CD25+ regulatory cells involves a granzyme B-dependent, perforin-independent mechanism. J. Immunol. 174, 1783–1786 15699103 10.4049/jimmunol.174.4.1783

[47] Bacchetta R., Bigler M., Touraine J. L., Parkman R., Tovo P. A., Abrams J., de Waal Malefyt R., de Vries J. E., Roncarolo M. G. (1994) High levels of interleukin 10 production in vivo are associated with tolerance in SCID patients transplanted with HLA mismatched hematopoietic stem cells. J. Exp. Med. 179, 493–502 7905018 10.1084/jem.179.2.493 PMC2191349

[48] Barrat F. J., Cua D. J., Boonstra A., Richards D. F., Crain C., Savelkoul H. F., de Waal-Malefyt R., Coffman R. L., Hawrylowicz C. M., O’Garra A. (2002) In vitro generation of interleukin 10-producing regulatory CD4(+) T cells is induced by immunosuppressive drugs and inhibited by T helper type 1 (Th1)- and Th2-inducing cytokines. J. Exp. Med. 195, 603–616 11877483 10.1084/jem.20011629 PMC2193760

[49] Veldman C., Höhne A., Dieckmann D., Schuler G., Hertl M. (2004) Type I regulatory T cells specific for desmoglein 3 are more frequently detected in healthy individuals than in patients with pemphigus vulgaris. J. Immunol. 172, 6468–6475 15128839 10.4049/jimmunol.172.10.6468

[50] Oh H. M., Yu C. R., Golestaneh N., Amadi-Obi A., Lee Y. S., Eseonu A., Mahdi R. M., Egwuagu C. E. (2011) STAT3 protein promotes T-cell survival and inhibits interleukin-2 production through up-regulation of Class O Forkhead transcription factors. J. Biol. Chem. 286, 30888–30897 21730069 10.1074/jbc.M111.253500 PMC3162449

[51] Iwasaki Y., Fujio K., Okamura T., Yanai A., Sumitomo S., Shoda H., Tamura T., Yoshida H., Charnay P., Yamamoto K. (2013) Egr-2 transcription factor is required for Blimp-1-mediated IL-10 production in IL-27-stimulated CD4+ T cells. Eur. J. Immunol. 43, 1063–1073 23349024 10.1002/eji.201242942

[52] Holter W., Fischer G. F., Majdic O., Stockinger H., Knapp W. (1986) T cell stimulation *via* the erythrocyte receptor. Synergism between monoclonal antibodies and phorbol myristate acetate without changes of free cytoplasmic Ca++ levels. J. Exp. Med. 163, 654–664 2419470 10.1084/jem.163.3.654 PMC2188057

[53] Meuer S. C., Hussey R. E., Fabbi M., Fox D., Acuto O., Fitzgerald K. A., Hodgdon J. C., Protentis J. P., Schlossman S. F., Reinherz E. L. (1984) An alternative pathway of T-cell activation: a functional role for the 50 kd T11 sheep erythrocyte receptor protein. Cell 36, 897–906 6231105 10.1016/0092-8674(84)90039-4

[54] Taylor A., Verhagen J., Akkoç T., Wenig R., Flory E., Blaser K., Akdis M., Akdis C. A. (2009) IL-10 suppresses CD2-mediated T cell activation *via* SHP-1. Mol. Immunol. 46, 622–629 18952289 10.1016/j.molimm.2008.07.031

[55] Bidère N., Briet M., Dürrbach A., Dumont C., Feldmann J., Charpentier B., de Saint-Basile G., Senik A. (2002) Selective inhibition of dipeptidyl peptidase I, not caspases, prevents the partial processing of procaspase-3 in CD3-activated human CD8(+) T lymphocytes. J. Biol. Chem. 277, 32339–32347 12080079 10.1074/jbc.M205153200

[56] Nguyen K. D., Vanichsarn C., Nadeau K. C. (2008) Increased cytotoxicity of CD4+ invariant NKT cells against CD4+CD25hiCD127lo/- regulatory T cells in allergic asthma. Eur. J. Immunol. 38, 2034–2045 18581330 10.1002/eji.200738082 PMC4217522

[57] Sharma V., Delgado M., Ganea D. (2006) Granzyme B, a new player in activation-induced cell death, is down-regulated by vasoactive intestinal peptide in Th2 but not Th1 effectors. J. Immunol. 176, 97–110 16365400 10.4049/jimmunol.176.1.97

[58] Che K. F., Shankar E. M., Muthu S., Zandi S., Sigvardsson M., Hinkula J., Messmer D., Larsson M. (2012) p38 Mitogen-activated protein kinase/signal transducer and activator of transcription-3 pathway signaling regulates expression of inhibitory molecules in T cells activated by HIV-1-exposed dendritic cells. Mol. Med. 18, 1169–1182 22777388 10.2119/molmed.2012.00103 PMC3510300

[59] Pot C., Jin H., Awasthi A., Liu S. M., Lai C. Y., Madan R., Sharpe A. H., Karp C. L., Miaw S. C., Ho I. C., Kuchroo V. K. (2009) Cutting edge: IL-27 induces the transcription factor c-Maf, cytokine IL-21, and the costimulatory receptor ICOS that coordinately act together to promote differentiation of IL-10-producing Tr1 cells. J. Immunol. 183, 797–801 19570826 10.4049/jimmunol.0901233 PMC2768608

[60] Apetoh L., Quintana F. J., Pot C., Joller N., Xiao S., Kumar D., Burns E. J., Sherr D. H., Weiner H. L., Kuchroo V. K. (2010) The aryl hydrocarbon receptor interacts with c-Maf to promote the differentiation of type 1 regulatory T cells induced by IL-27. Nat. Immunol. 11, 854–861 20676095 10.1038/ni.1912 PMC2940320

[61] Szymczak A. L., Workman C. J., Wang Y., Vignali K. M., Dilioglou S., Vanin E. F., Vignali D. A. (2004) Correction of multi-gene deficiency in vivo using a single ‘self-cleaving’ 2A peptide-based retroviral vector. Nat. Biotechnol. 22, 589–594 15064769 10.1038/nbt957

[62] Andolfi G., Fousteri G., Rossetti M., Magnani C. F., Jofra T., Locafaro G., Bondanza A., Gregori S., Roncarolo M. G. (2012) Enforced IL-10 expression confers type 1 regulatory T cell (Tr1) phenotype and function to human CD4(+) T cells. Mol. Ther. 20, 1778–1790 22692497 10.1038/mt.2012.71 PMC3437582

