# Supplementary material for: STAT3 governs hyporesponsiveness and granzyme B-dependent suppressive capacity in human CD4+ T cells
Source: FASEB J. 2014 Nov 14;29(3):759–71. doi: 10.1096/fj.14-257584 (PMC4422363; doi:10.1096/fj.14-257584)
Supplement: Supplemental Data [file supp_29_3_759__index.html]

STAT3 governs hyporesponsiveness and granzyme B-dependent suppressive capacity in human CD4+ T cells — STAT3 governs hyporesponsiveness and granzyme B-dependent suppressive capacity in human CD4+ T cells — Supplemental Data 

# STAT3 governs hyporesponsiveness and granzyme B-dependent suppressive capacity in human CD4+ T cells

## Supplemental Data

**Files in this Data Supplement:**

- Supplemental Data
- Supplemental Data
- Supplemental Data
- Supplemental Data
