# Supplementary material for: STAT3 governs hyporesponsiveness and granzyme B-dependent suppressive capacity in human CD4+ T cells
Source: FASEB J. 2014 Nov 14;29(3):759–71. doi: 10.1096/fj.14-257584 (PMC4422363; doi:10.1096/fj.14-257584)
Supplement: Supplemental Data [file supp_fj.14-257584_Supplemental_Figure1.pdf]

Supplemental Figure S1

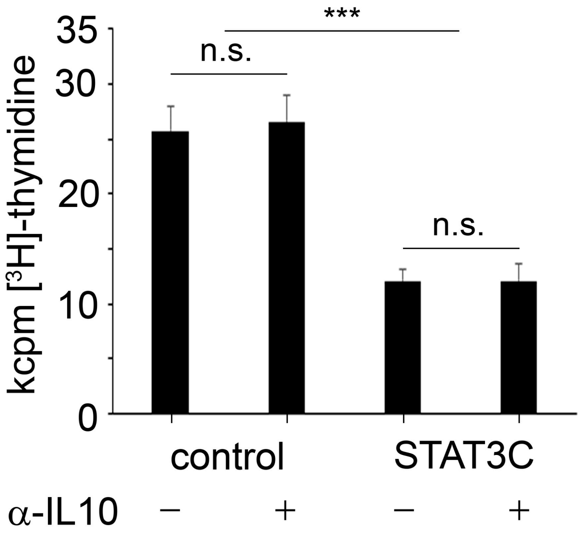

SUPPLEMENTAL FIGURE S1. STAT3C-mediated hyporesponsiveness is independent of IL-10 production. Proliferation of control-vector or STAT3C-transduced CD4$^+$ T-cells in response to anti-CD3/anti-CD28 stimulation in the absence or presence of an IL-10 neutralizing antibody (20 μg/mL). Mean proliferation + SD from one representative donor (n=3) are shown; n.s. not significant, *** $p<0.001$
