# Supplementary material for: STAT3 governs hyporesponsiveness and granzyme B-dependent suppressive capacity in human CD4+ T cells
Source: FASEB J. 2014 Nov 14;29(3):759–71. doi: 10.1096/fj.14-257584 (PMC4422363; doi:10.1096/fj.14-257584)
Supplement: Supplemental Data [file supp_fj.14-257584_Supplemental_Figure2.pdf]

Supplemental Figure S2

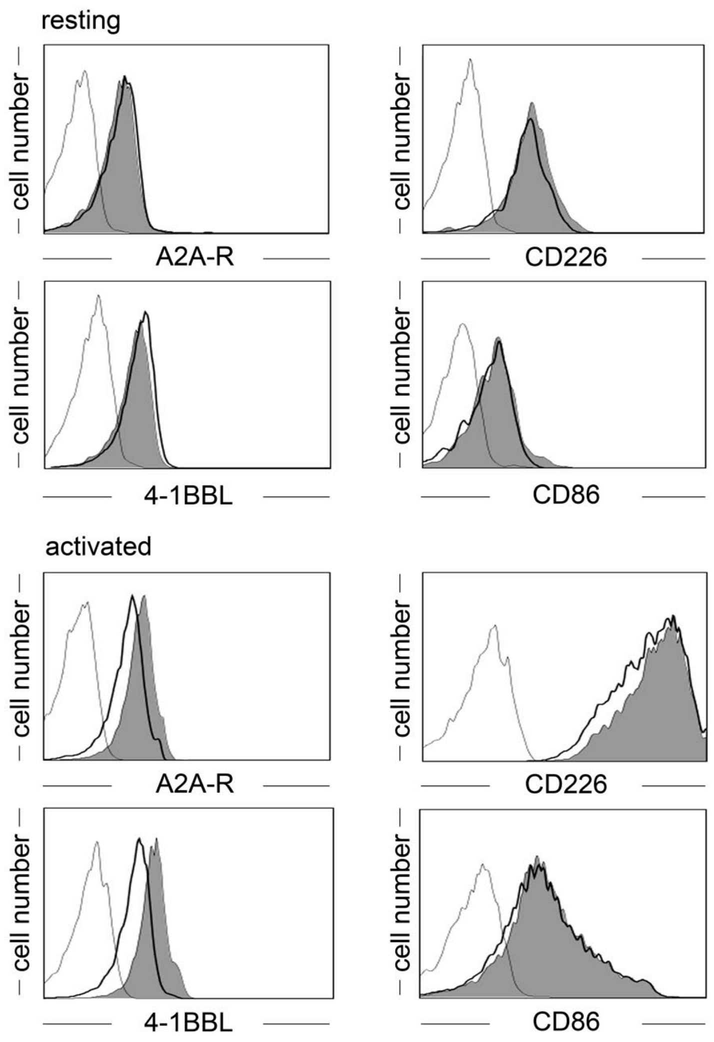

SUPPLEMENTAL FIGURE S2. STAT3C^{+} T-cells do not show up-regulation of Tr1-associated surface molecules. Surface expression of the indicated molecules of STAT3C-transduced (black line) or control-vector transduced T-cells (grey histograms) either in a resting state or 24 hours after anti-CD3/anti-CD28 stimulation. Fine grey lines represent staining with isotype-matched control antibodies. One representative experiment out of four is depicted.
