# Supplementary material for: STAT3 governs hyporesponsiveness and granzyme B-dependent suppressive capacity in human CD4+ T cells
Source: FASEB J. 2014 Nov 14;29(3):759–71. doi: 10.1096/fj.14-257584 (PMC4422363; doi:10.1096/fj.14-257584)
Supplement: Supplemental Data [file supp_fj.14-257584_Supplemental_Figure3.pdf]

Supplemental Figure S3

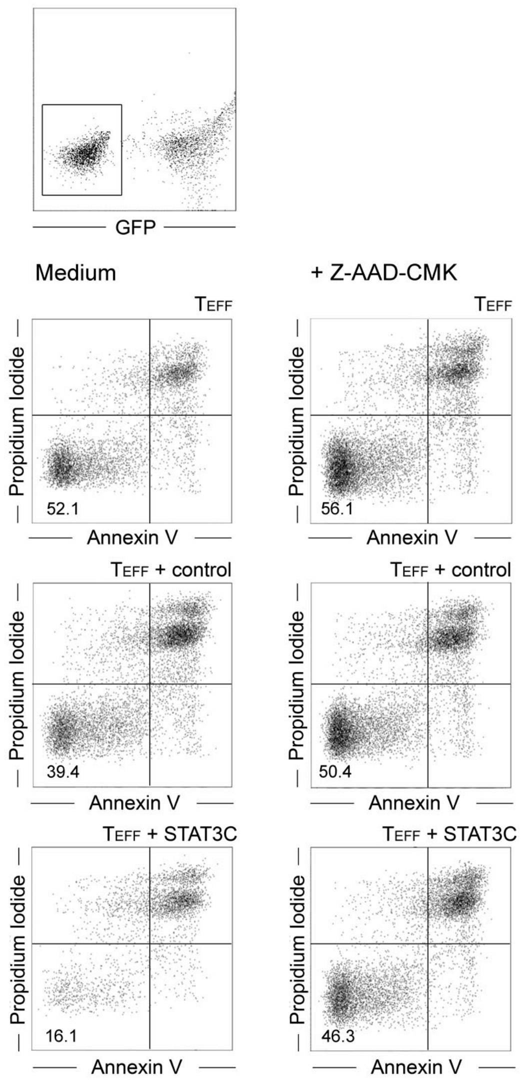

SUPPLEMENTAL FIGURE S3. Granzyme B-dependent induction of cell death conferred by STAT3C$^+$ T-cells. Viability of GFP-negative T_{EFF} was determined upon coculture with the indicated T-cells in the absence (left panel) or presence (right panel) of the granzyme B inhibitor Z-AAD-CMK by flow cytometry by quantification of cells negative for annexin V and propidium iodide. Numbers indicate percentage of viable cells in respective quadrant. Figure shows one representative experiments out of 6 performed.
