# Supplementary material for: STAT3 governs hyporesponsiveness and granzyme B-dependent suppressive capacity in human CD4+ T cells
Source: FASEB J. 2014 Nov 14;29(3):759–71. doi: 10.1096/fj.14-257584 (PMC4422363; doi:10.1096/fj.14-257584)
Supplement: Supplemental Data [file supp_fj.14-257584_Supplemental_Table1.docx]

**Supplemental Data Table I. List of antibodies used in this study**

| Specificity | Clone | Species | Conjugated fluorochrome^a)^ | Supplier |
| --- | --- | --- | --- | --- |
| CD3 | OKT3 | mouse | eFluor450 | eBioscience, San Diego, CA |
| CD4 | L200 | mouse | V500/FITC | BD, Franklin Lakes, NJ |
| CD25 | BC96 | mouse | APC-eFluor780 | eBioscience, San Diego, CA |
| CD39 | eBioA1 | mouse | PerCP-eFluor710 | eBioscience, San Diego, CA |
| CD45RA | 5H9 | mouse | APC-H7 | BD, Franklin Lakes, NJ |
| CD49b | AK-7 | mouse | AlexaFluor488/unconjugated | Biolegend, San Diego, CA |
| CD86 | IT2.2 | mouse | APC | Biolegend, San Diego, CA |
| CD127 | eBioRDR5 | mouse | APC | eBioscience, San Diego, CA |
| CD152/CTLA-4 | 14D3 | mouse | PE | eBioscience, San Diego, CA |
| CD226 | 11A8 | mouse | PE | Biolegend, San Diego, CA |
| LAG3 | Polyclonal | goat | APC | RnD Systems, Minneapolis, MN |
| 4-1BBL | 5F4 | mouse | PE | Biolegend, San Diego, CA |
| A2A-R | 7F6-G5-A2 | mouse | PerCP-Cy5.5 | Santa Cruz, Dallas, TX |
| Granzyme A | CB9 | mouse | Pacific Blue | Biolegend, San Diego, CA |
| Granzyme B | GB11 | mouse | AlexaFluor647 | Biolegend, San Diego, CA |
| STAT3 | 232209 | mouse | PE | RnD Systems, Minneapolis, MN |
| p-STAT3 (pY705) | 4/P-STAT3 | mouse | PerCP-Cy5.5 | BD, Franklin Lakes, NJ |
| FOXP3 | PCH101 | mouse | APC | eBioscience, San Diego, CA |
| Mouse IgG |  | goat | PE | Jackson, Bar Harbor, ME |

^a)^ Abbreviations: APC, allophycocyanin; FITC, fluorescein isothiocyanate; Cy, cyanine; PE, phycoerythrin; PerCP, peridinin chlorophyll; PerCP-Cy5.5, peridinin chlorophyll-cyanine;
